# Evaluation of glasses‐free 3D anatomy learning materials through a randomized control study with a puzzle‐like method

**DOI:** 10.1002/ase.70063

**Published:** 2025-06-01

**Authors:** Satoru Muro, Keisuke Miyosawa, Kumiko Yamaguchi, Kentaro Okamoto, Shota Okamoto, Tomoki Itamiya, Keiichi Akita

**Affiliations:** ^1^ Department of Clinical Anatomy, Graduate School of Medical and Dental Sciences Institute of Science Tokyo Bunkyo‐ku Tokyo Japan; ^2^ Center for Innovative Teaching and Learning Institute of Science Tokyo Meguro‐ku Tokyo Japan; ^3^ Department of Specialized Surgeries, Graduate School of Medical and Dental Sciences Institute of Science Tokyo Bunkyo‐ku Tokyo Japan; ^4^ Department of Dental Education, School of Dentistry Kanagawa Dental University Yokosuka Kanagawa Japan

**Keywords:** 3D visualization, anatomy learning materials, glasses‐free 3D display, learning outcomes, medical education, randomized controlled trial

## Abstract

3D visualization tools have been developed to assist in the anatomical learning of medical students, but the evidence on the effectiveness of these tools is inconsistent. Conventional 3D materials are often displayed on 2D screens, which can limit their educational impact. This study evaluates the effectiveness of the presentation of 3D learning materials using 3D displays compared to 2D displays in a randomized controlled trial. We developed glasses‐free 3D learning materials for the anatomy of abdominal organs and blood vessels, which are compatible with the 3D spatial reality tabletop display EFL‐SR1 developed by Sony. We assessed the effectiveness of these materials in a randomized controlled trial. Fifty‐six medical students were randomly divided into two groups to learn using either 2D or 3D displays. The improvement in understanding of the spatial arrangement of anatomical structures was measured before and after the learning sessions using a novel 3D puzzle‐like method. Both groups demonstrated improvements in understanding of the spatial arrangement of anatomical structures; there were no significant differences in overall performance between the 2D and 3D display groups. The 3D display group exhibited less variability in improvement indices, suggesting more consistent learning outcomes. The study indicates that glasses‐free 3D displays can minimize learning disparities among medical students and provide a more universally effective learning environment for all students. The 3D learning materials compatible with glasses‐free 3D displays and the innovative 3D puzzle‐like method used in this study offer a novel approach to teaching and evaluating anatomy education.

## INTRODUCTION

Understanding the spatial arrangement of organs, muscles, nerves, and vessels is essential for human anatomy learning and, concomitantly, is one of medical students' most daunting learning tasks. In recent years, learning materials using three‐dimensional (3D) visualization, such as applications that allow users to view 3D human body models, have been developed and increased in importance.^1^, ^2^, ^3^ Digital human anatomy platforms, some of which are available as mobile applications, have gained widespread use and served as alternatives for anatomy education during the COVID‐19 pandemic.^4^, ^5^, ^6^, ^7^ Such platforms include Visible Body, Complete Anatomy, Primal Pictures, Anatomy Learning, Z Anatomy, and 3D Atlas Human Embryology. The effectiveness of these 3D applications in anatomy education has been evaluated in recent years.^8^, ^9^, ^10^, ^11^ Several randomized controlled trials (RCTs) have compared the learning outcomes of 3D applications with those of traditional methods such as textbooks, atlases, plastic models, and cadaveric dissection. While some studies have reported significantly higher learning outcomes with 3D applications,^12^, ^13^, ^14^, ^15^, ^16^ others found no significant differences in knowledge acquisition.^17^, ^18^, ^19^, ^20^, ^21^ Even in cases where no significant differences in learning outcomes were observed, some studies reported that 3D applications significantly enhanced students' satisfaction, enjoyment, and motivation to learn.^22^, ^23^ Separate from these 3D applications, some of which operate on common devices such as PCs and mobile devices, the Anatomage Table—a 3D application implemented on a table‐shaped monitor—has been evaluated as a complementary tool for anatomy education.^24^, ^25^, ^26^, ^27^, ^28^ In an RCT involving medical students, the group that used the Anatomage Table scored significantly higher on tests for specific learning items (e.g., the radius) compared to the group using traditional methods; however, no significant differences were observed for other learning items.^29^ Another RCT reported a trend of higher pass rates for students using the Anatomage Table compared to those studying with traditional textbooks, although the difference was not statistically significant.^30^ In addition, learning methods incorporating stereoscopy, virtual reality (VR), or augmented reality (AR) have been developed to enhance the effectiveness of 3D content.^31^, ^32^ However, evaluations of these methods proved inconsistent. Some RCTs reported significantly better learning outcomes than monoscopic learning, 2D materials, atlases, or cadaveric dissections,^33^, ^34^, ^35^, ^36^, ^37^ while others found no significant differences.^38^, ^39^, ^40^, ^41^, ^42^ Thus, while the effectiveness of learning methods utilizing 3D digital applications remains an area of ongoing research, these tools are gaining attention as promising complementary resources for anatomy education.

We believe there are at least two limitations to using these 3D models in human anatomy learning. First, the limitation of the display method. Despite the content being in 3D, most 3D anatomy learning materials are displayed in 2D on a regular flat display,^15^, ^21^, ^23^, ^34^ which may not maximize the learning effect of the 3D content. This mismatch between spatially rich 3D content and 2D visualization has been highlighted as a challenge for learners attempting to mentally transition between dimensions.^43^ One way to achieve actual 3D viewing is through virtual reality (VR) or augmented reality tools, which are beginning to be used for 3D anatomy learning.^33^, ^39^ However, VR‐ and augmented reality–based learning requires wearing VR headsets, and these devices have particular issues when used for anatomy education, including physical fit (e.g., individual's head size and shape and whether it requires wearing glasses) and discomfort (e.g., dizziness, nausea, motion sickness, and fatigue) that vary by student. To tackle this problem, the current study focused on using the 3D spatial reality tabletop display (hereinafter just 3D display) EFL‐SR1 developed by Sony; this tabletop display recognizes the user's line of sight, projecting images according to the left and right eyes and allowing the user to experience autostereoscopic (i.e., glasses‐free) 3D viewing – in other words, to view 3D images with the naked eye. Such devices hold the potential to become more common in the near future (e.g., similar to the current situation for VR tools) and to make autostereoscopic vision more accessible, thereby allowing the realization of anatomy learning materials that can be more easily viewed in 3D on a tabletop.

Second, there are limitations associated with the methods used to evaluate the learning effectiveness of 3D learning materials. There is a host of literature on the effectiveness of 3D learning materials, but their conclusions remain inconsistent^44^; for example, while some described 3D as being more effective than 2D,^20^, ^34^ others found no significant difference.^23^, ^38^, ^42^ One of the factors contributing to this inconsistency may be the learning assessment methodology used. The advantage of 3D over 2D learning materials (e.g., anatomical atlases) is that they give a 3D impression with more depth, making it easier to understand the spatial arrangement of anatomical structures. Most previous studies have used paper tests with multiple‐choice and/or word‐answer questions to assess learning effects, which may fall short of thoroughly evaluating the characteristics and learning effects of 3D learning materials.^15^, ^16^, ^23^, ^38^, ^39^, ^45^ Therefore, to directly assess how learners understand the spatial arrangement of organs after engaging with 3D learning materials, we deemed it more appropriate to ask learners to place organs in a 3D virtual space, resembling the processes used in puzzles, and quantitatively evaluate the accuracy of their placements.

This study endeavored to address these problems related to the currently‐used display and evaluation methods for 3D learning materials, having two main objectives: (1) develop 3D anatomy learning materials for use with a 3D display and (2) introduce a method for more directly measuring the learning effects of 3D learning materials on understanding of the spatial arrangement of anatomical structures—defined here as the comprehension of how anatomical structures are arranged in three‐dimensional space relative to one another—and comparing the effectiveness of a 3D display with that of a 2D display in an RCT. Clarifying the characteristics and effectiveness of 3D displays stands to contribute to the dissemination of 3D anatomy learning materials, the construction of optimal 3D learning material utilization methods, and the development of anatomy education.

This study was guided by three key educational theories: constructivism, dual coding theory, and cognitive load theory. Constructivism emphasizes building new knowledge on prior knowledge, which is essential in anatomy where understanding spatial relationships is critical. Dual coding theory supports the integration of visual and verbal information to enhance learning. Cognitive load theory underlines the importance of reducing unnecessary mental effort to facilitate effective learning. These theoretical foundations informed the design of the 3D display learning materials and the puzzle‐based evaluation approach adopted in this study.

### Study goals

This study aimed to develop 3D anatomy learning material for 3D displays, introduce a novel evaluation method to directly measure learning effectiveness, and compare the effectiveness of 3D displays with 2D displays through an RCT. The research question was: Does learning using a 3D display lead to greater learning effectiveness than learning using a 2D display? The hypothesis posits that learning about anatomy using 3D digital learning materials displayed in 3D improves the understanding of the spatial arrangement of anatomical structures more effectively than learning in a 2D display environment. This study adopted a positivist approach to quantitatively measure learning effectiveness and perform comparisons and evaluations based on the measured data.

## MATERIALS AND METHODS

### Development of the 3D anatomy learning materials

#### Creation of 3D objects of abdominal organs and blood vessels

We used data from the anatomical sections of the Visible Korean Human open resource (33 years old, male; http://anatomy.co.kr/) and publicly‐available, manually‐segmented data for the 3D reconstruction of abdominal organs and blood vessels.^46^, ^47^, ^48^, ^49^ By modifying the publicly available segmented data, including 431 abdominal images, the abdominal arteries, veins, stomach, duodenum, cecum, appendix, colon, rectum, kidneys, bladder, liver, gallbladder, and pancreas were segmented. The 3D reconstruction was performed using TRI/3DSRFII (version R.12.00.02.6‐H; Ratoc, Tokyo, Japan; http://www.ratoc.com/eng/).

#### Development of 3D anatomical learning materials compatible with a 3D display

**FIGURE 1:**
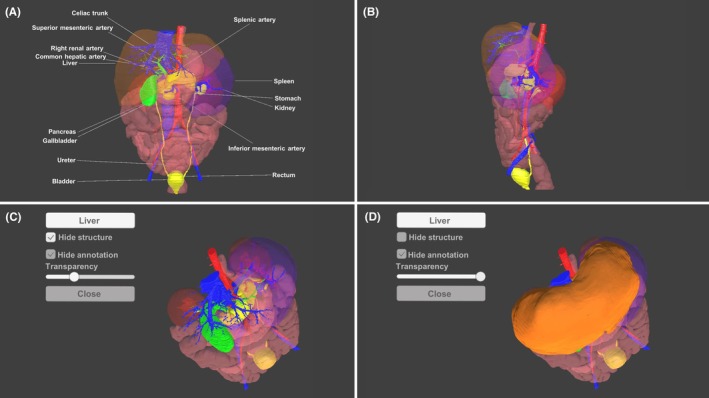
3D anatomical learning materials developed in this study. (A) Detailed 3D representations of abdominal organs and blood vessels, each labeled with their respective anatomical structures. (B) Interactive features allowing for the rotation of the 3D objects in the horizontal, sagittal, and frontal planes. Additional features include zooming in/out and moving the 3D objects for a comprehensive view. (C) A feature that enables toggling the visibility of each organ for a focused study of individual structures. (D) An option to change the transparency of each organ, providing a unique perspective on the spatial relationships between different anatomical structures.

The 3D anatomy learning materials utilized in this study were developed using Unity (version 2020.3.48f1, Unity Technologies) to be compatible with a 3D display. The learning material displays 3D objects of abdominal organs and blood vessels on the screen and provides features essential for learning (e.g., structure labeling, rotation, zoom in/out, the possibility of moving the 3D objects, toggling the visibility of each organ, and changing the transparency of each organ) (Figure 1). The software learning material that was developed supports the EFL‐SR1 (Sony) 3D display and is controlled via the GameSir T4w PC controller (GameSir, China).

### Evaluation of the effectiveness of the 3D display

#### Study design and settings

An RCT was conducted to evaluate the effectiveness of the 3D display. Participants were randomly divided into 2D and 3D display groups, with 28 participants in each group. Adaptive randomization was employed, meaning that each time a participant was enrolled in the trial, randomization was performed considering the balance of the existing groups. This approach was taken to minimize differences in medical student academic year distribution across the groups.

**FIGURE 2:**
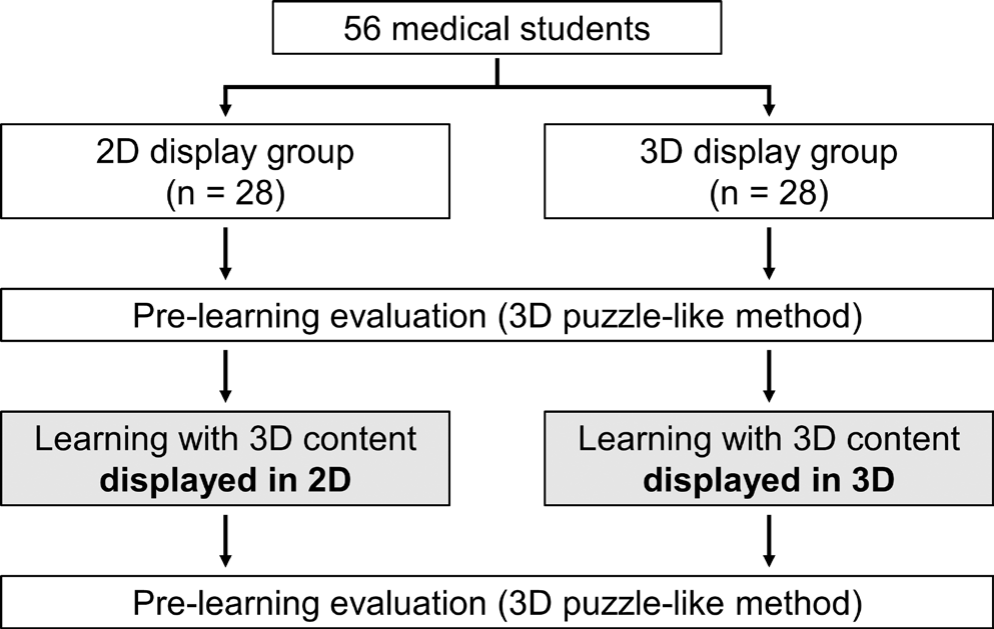
Schematic representation of the randomized controlled trial design. The trial aimed to evaluate the effectiveness of the different display methods on learning outcomes among 56 medical students. Students were randomly assigned to either the 2D or the 3D display group, with 28 students in each group. Initially, both groups underwent a pre‐learning evaluation using a 3D puzzle‐like method to assess their baseline knowledge/skills. The 2D display group then engaged in a learning session with 3D content presented in a 2D display, while the 3D display group engaged in a learning session with 3D content presented in a glasses‐free 3D display. Upon completion of the learning session, both groups were re‐evaluated using the same 3D puzzle‐like method used in the pre‐learning evaluation to determine the impact of the display format on their understanding of the spatial arrangement of anatomical structures.

**FIGURE 3:**
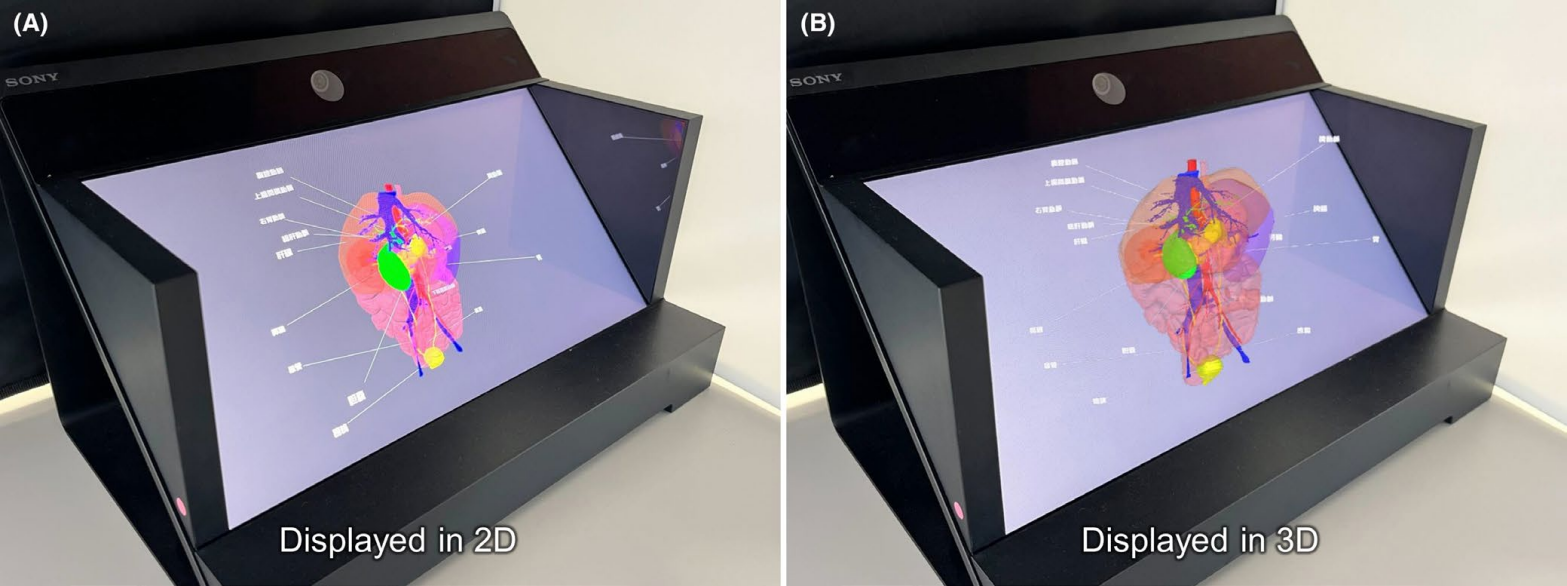
Two different display modalities used in the study. (A) The 3D anatomical learning materials developed in this study are presented in a 2D display, where the anatomical structures are represented on a flat display. Please note that this is an illustrative diagram, not a photograph. (B) The 3D anatomical learning materials developed in this study are presented in a glasses‐free 3D display, where the anatomical structures appear to have depth and volume, rendering the representation more realistic. Please note that this is an illustrative diagram, not a photograph.

Each group underwent a series of tasks, namely pre‐learning evaluation, learning, and post‐learning evaluation tasks (Figure 2). In the learning task, the 2D display group viewed the developed 3D learning material using the 2D display function of the EFL‐SR1 (Sony) (Figure 3A), as it also offers a 2D display similar to a conventional 2D display. The 3D display group viewed the learning material in 3D through the EFL‐SR1 3D display (Sony) (Figure 3B). The learning software allowed the enablement of 360° rotation, depending on the settings. However, the settings were adjusted to allow only horizontal rotation when conducting the RCT. This was done to leverage the advantages of 3D digital content while simplifying the operation to reduce the burden on participants. Since the RCT was conducted within a limited learning time frame, this adjustment was designed to minimize the influence of participants' varying levels of familiarity or proficiency with the software. The creation of a simpler operational environment was intended to reduce the time spent on learning how to use the software and increase the proportion of time spent on actual learning within the designated session. Additionally, to minimize the potential impact of participant fatigue or loss of focus on learning and evaluation, the total duration of the study—including the explanation and obtaining of consent, pre‐learning assessments, learning time, and post‐learning assessments—was targeted to remain within approximately 30 min. Specifically, the learning time was set at three minutes, and each group was allowed to view the learning material for three minutes.

#### Study participants

In total, 56 1st–6th‐year medical students enrolled in the Tokyo Medical and Dental University were included in the study to examine trends specific to this university. Regarding the use of a single university as the setting, we needed to verify the results in a sample of medical students with similar educational backgrounds; since the curricula vary between universities, we deemed it preferable to include only one university to secure greater similarity regarding educational background. Therefore, this study assumed that Tokyo Medical and Dental University medical students were the target population. Additionally, participants were recruited from various academic years because each year's intake at the university has only about 100 students, and securing a sample size sufficient for the study required recruiting volunteer medical students from all years. Regarding sampling methodology, we requested participation from students in the relevant academic years through face‐to‐face interactions and emails, and those who agreed to participate were entered into the study. The procedure for registration in the study involved obtaining consent and registering participation using WebClass, the university's learning management system.

In Japan, medical education follows a 6‐year program. At Tokyo Medical and Dental University, anatomy classes, comprising approximately 152 slots, are scheduled during the first semester of the second year of the medical course. During the second year, cadaver‐based dissection practice is conducted. This study was conducted in the second semester of 2023 when first‐year students had not taken anatomy classes, whereas students from the second to the sixth years had already completed them.^50^ Additionally, it should be noted that, according to the curriculum, not all students had used the virtual materials (e.g., 3D resources) directly related to this study.

To ensure adequate power for detecting a significant difference between the groups regarding the improvement index, a sample size calculation was performed with a confidence interval of 95% (*α* = 0.05) and a standard deviation of 0.15. Considering an expected mean difference between the groups of 0.12, a standard deviation of 0.15, a significance level (*α*) of 0.05, and a desired power (1‐*β*) of 0.8, and assuming equal sample sizes for each group, the effect size was calculated to be 0.8. Then, using the TTestIndPower function from the statsmodels.stats.power module in Python, we calculated the sample size for each group, yielding 25 participants. For a sample size of 25 participants per group, the margin of error was calculated to be approximately 0.059. Since there were 28 participants in each group, this study secured an appropriate sample size to guarantee adequate power to detect significant differences between the groups under the specified conditions.

#### 3D puzzle activity

**FIGURE 4:**
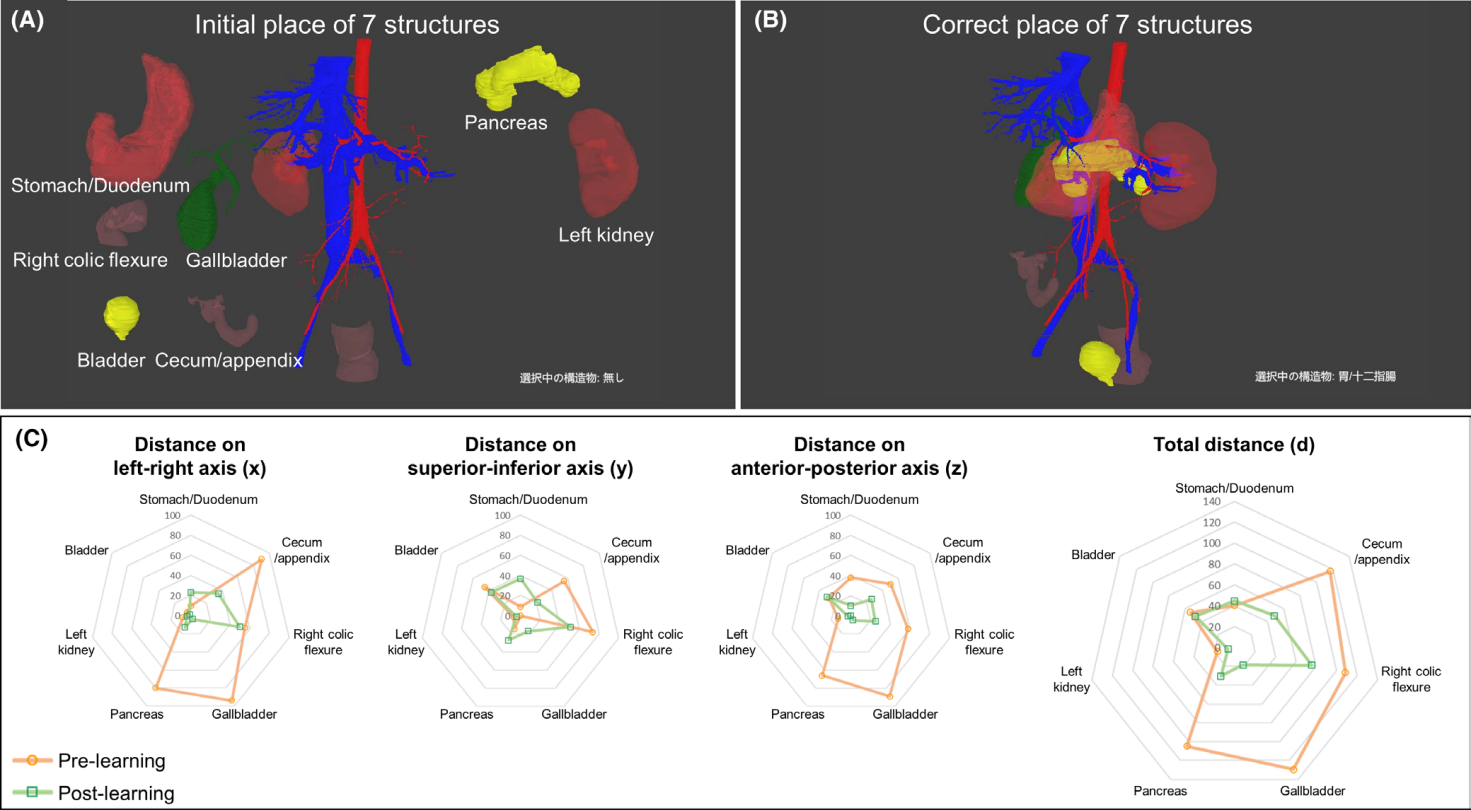
Evaluation of the understanding of the spatial arrangement of anatomical structures using a 3D puzzle‐like method. (A) Initial placement of the seven anatomical structures in the software interface. Users move each organ within the 3D space to a position they deem correct. (B) The correct placement of the seven structures for comparison. The differences (distances) between the user's placements and the correct positions were measured. (C) Radar charts depicting the distances between the participants' placement of each structure, the correct position on the three axes, and the total distance before and after the learning intervention. A closer position to the center of the radar chart signifies a more accurate organ placement and a superior comprehension of anatomical structures' spatial arrangement. Placements before learning are marked with an orange line, while placements after learning are denoted with a green square line.

Both the 2D and 3D display groups completed the same pre‐learning and post‐learning evaluation tasks. These tasks involved the use of a software program that allowed organs in the 3D learning material to be arranged like a puzzle, enabling the assessment of the understanding of the spatial arrangement of anatomical structures of students regarding the arrangement of the relevant structures. This 3D puzzle‐like method was developed by modifying the 3D learning material we had developed, allowing users to freely move seven structures—the stomach/duodenum, cecum/appendix, right colic flexure, gallbladder, pancreas, left kidney, and bladder—with fixed blood vessels over a 3D space (Figure 4A,B). Participants were asked to move the structures, initially placed in a separate location, to positions they considered correct according to human anatomy.

Understanding of the spatial arrangement of anatomical structures, specifically the comprehension of how anatomical structures are arranged three‐dimensionally, has been evaluated in many past studies using paper tests (e.g., multiple‐choice and/or word‐answer questions).^40^, ^41^, ^44^, ^45^, ^46^, ^49^ As mentioned in the Introduction, we believe that paper tests have limitations in accurately measuring understanding of the spatial arrangement of anatomical structures, which is one of the key issues addressed in this study. While 3D digital content is often utilized in the learning phase, the evaluation phase still predominantly relies on 2D methods. This mismatch between educational tools and evaluation methods has also been highlighted in previous studies.^40^ However, no method has yet been established to directly and quantitatively assess students' understanding of the spatial arrangement of anatomical structures. We adopted a novel evaluation approach different from traditional paper tests to address this gap. Specifically, we utilized a unique puzzle‐like method designed to directly evaluate the understanding of the spatial arrangement of anatomical structures.

#### Measurements and evaluations

The position of the structures was defined by the centroid of each structure, and the absolute differences in coordinates between the participants' placements and the correct positions for each structure were output in the form of $\left(x,y,z\right)$ in a 3D Cartesian coordinate system (x≥0,y≥0,z≥0). This was then converted to a distance d ($\equiv \sqrt{x^{2}+y^{2}+z^{2}}$). In this coordinate system, “1” was equal to 1 mm, with the *x*‐axis representing the left–right axis of the body, the *y*‐axis the superior–inferior axis, and the *z*‐axis the anterior–posterior axis. Values of x, y, z, and d closer to 0 indicated an organ positioned closer to its correct location, suggesting a better understanding of the spatial arrangement of anatomical structures. The understanding of the spatial arrangement of anatomical structures of participants was visualized in radar charts for both the pre‐learning and post‐learning evaluation tasks (Figure 4C), where positions closer to the center of the chart indicated an organ positioned closer to its correct location.

For each participant, the sums of x, y, z, and d for the seven structures were calculated and denoted as X, Y, Z, and D, respectively. For the pre‐learning evaluation, the values were denoted as $X_{\mathrm{pre}}$, $Y_{\mathrm{pre}}$, $Z_{\mathrm{pre}}$, and $D_{\mathrm{pre}}$; for the post‐learning evaluation, they were denoted as $X_{\text{post}}$, $Y_{\text{post}}$, $Z_{\text{post}}$, and $D_{\text{post}}$. These values respectively indicated the understanding of the spatial arrangement of anatomical structures in the left–right direction, the superior–inferior direction, the anterior–posterior direction, and total distance.

These improvement indices were designed to quantify how much each participant's accuracy in identifying the spatial positions of anatomical structures changed as a result of the learning intervention. Their purpose was to capture learning gains in a direction‐specific and individualized manner, which allows for more detailed analysis than relying solely on average scores. The improvement indices $I_{X}$, $I_{Y}$, $I_{Z}$, and $I_{D}$ were then calculated for each individual, as follows:

$$I_{D}\equiv \frac{D_{\mathrm{pre}}-D_{\text{post}}}{D_{\text{post}}},I_{X}\equiv \frac{X_{\mathrm{pre}}-X_{\text{post}}}{X_{\text{post}}},I_{X}\equiv \frac{X_{\mathrm{pre}}-X_{\text{post}}}{X_{\text{post}}},I_{Z}\equiv \frac{Z_{\mathrm{pre}}-Z_{\text{post}}}{Z_{\text{post}.}}$$

#### Statistical analysis

The improvement indices were compared between the 2D and 3D display groups. For statistical analysis, a graphical user interface for R (The R Foundation for Statistical Computing, Vienna, Austria) named EZR (version 1.61, Saitama Medical Center, Jichi Medical University) was used.^45^ To compare the understanding of the spatial arrangement of anatomical structures pre‐ and post‐learning between the 2D and 3D display groups, a Mann–Whitney *U* test was conducted. A one‐sided *F*‐test was performed to compare improvement index variations between the two groups.

### Ethical approval

This study was approved by the Ethics Committee of Tokyo Medical and Dental University (Approval number: C2023‐024) and conducted in accordance with the 1964 Helsinki Declaration and its later amendments. All participants provided informed consent in person and electronically through the university's learning management system (WebClass). Information about the study, including the right to withdraw, was posted on WebClass to allow participants to reconsider their participation at any time.

To ensure fairness, both the 2D and 3D display groups were provided with the same learning content and objectives. The study design, which intentionally used different visualization methods, aimed to assess their educational impact without disadvantaging any participants. Assessment criteria were standardized across groups. Participant anonymity was preserved through the use of unique identifiers, with no personal information collected. All data were stored securely and will be retained for five years in accordance with institutional guidelines.

## RESULTS

The characteristics of the medical student participants are listed in Table 1. The students were randomly divided into the two groups to ensure a balanced representation of students from different academic years. Upon evaluating the participants' understanding of the spatial arrangement of anatomical structures before and after learning, both the 2D and the 3D display groups showed improvements in $D_{\text{post}}$ compared to $D_{\mathrm{pre}}$ (2D group: $D_{\mathrm{pre}}\;450.01\pm 156.88$, $D_{\text{post}}\;275.55\pm 101.96$; 3D group: $D_{\mathrm{pre}}\;418.79\pm 134.38$, $D_{\text{post}}\;251.80\pm 119.53$). In both groups, $D_{\text{post}}$ values were significantly lower than $D_{\mathrm{pre}}$ values, indicating a significant improvement in understanding of the spatial arrangement of anatomical structures after learning (Mann–Whitney *U* test, 2D group: $p=3.27\times 10^{-5}$, 3D group: $p=1.51\times 10^{-6}$). No differences were observed between the two groups in either the pre‐learning or post‐learning evaluations (Mann–Whitney *U* test, $D_{\mathrm{pre}}:p=0.62$, $D_{\text{post}}:p=0.332$) (Table 2).

**TABLE 1:** Participants' characteristics.

	2D display group (*n* = 28)	3D display group (*n* = 28)
Gender, *n* (%)		
Men, *n* (%)	14 (50.0%)	17 (60.7%)
Women, *n* (%)	14 (50.0%)	11 (39.3%)
Age, mean (±standard deviation)	22.1 (±3.6)	21.3 (±1.8)
Academic year		
First year, *n* (%)	4 (14.3%)	4 (14.3%)
Second year, *n* (%)	6 (21.4%)	5 (17.9%)
Third year, *n* (%)	3 (10.7%)	4 (14.3%)
Fourth year, *n* (%)	6 (21.4%)	6 (21.4%)
Fifth year, *n* (%)	5 (17.9%)	5 (17.9%)
Sixth year, *n* (%)	4 (14.3%)	4 (14.3%)

*Note*: Table 1 presents the characteristics of participants in the study, categorized into the 2D display group (*n* = 28) and the 3D display group (*n* = 28). The table includes a breakdown of gender distribution (men and women), mean age with standard deviation, and academic year classification (first through sixth year). Percentages are provided in parentheses for each subgroup.

**TABLE 2:** Mean of improvement indices and standard deviations for each group.

	2D display group	3D display group	*p*‐value (comparing distributions)	*p*‐value (comparing variances)
$D_{\mathrm{pre}}$	450.01 (±156.88)	418.79 (±134.38)	0.62	0.426
$D_{\text{post}}$	275.55 (±101.96)	251.80 (±119.53)	0.332	0.414
*p‐*value ($D_{\mathrm{pre}}$ vs. $D_{\text{post}}$)	$3.27\times 10^{-5}$*	$1.51\times 10^{-6}$*		
Total ($I_{D}$)	0.37 (±0.21)	0.39 (±0.14)	0.248	0.022*
Left–right axis ($I_{X}$)	0.25 (±0.74)	0.35 (±0.43)	0.298	0.003*
Superior–inferior axis ($I_{Y}$)	0.41 (±0.21)	0.44 (±0.18)	0.980	0.197
Anterior–posterior axis ($I_{Z}$)	0.10 (±0.55)	0.25 (±0.32)	0.107	0.003*

*Note*: Table 2 summarizes the mean values and standard deviations for improvement indices in the 2D display group and 3D display group, respectively. The indices include: $D_{\mathrm{pre}}$: Pre‐learning evaluation results; $D_{\text{post}}$: Post‐learning evaluation results; $I_{D}$: Total improvement index, combining all axes; $I_{X}$: Improvement index along the left–right axis; $I_{Y}$: Improvement index along the superior–inferior axis; $I_{Z}$: Improvement index along the anterior–posterior axis. The table includes *p*‐values for: *p‐*value (comparing distributions): Statistical significance of differences in the distributions between the two groups, calculated using the Mann–Whitney *U* test; *p‐*value (comparing variances): Statistical significance of differences in variances between the two groups, calculated using a one‐sided *F*‐test. Values marked with an asterisk (*) indicate statistically significant differences (*p* < 0.05).

**FIGURE 5:**
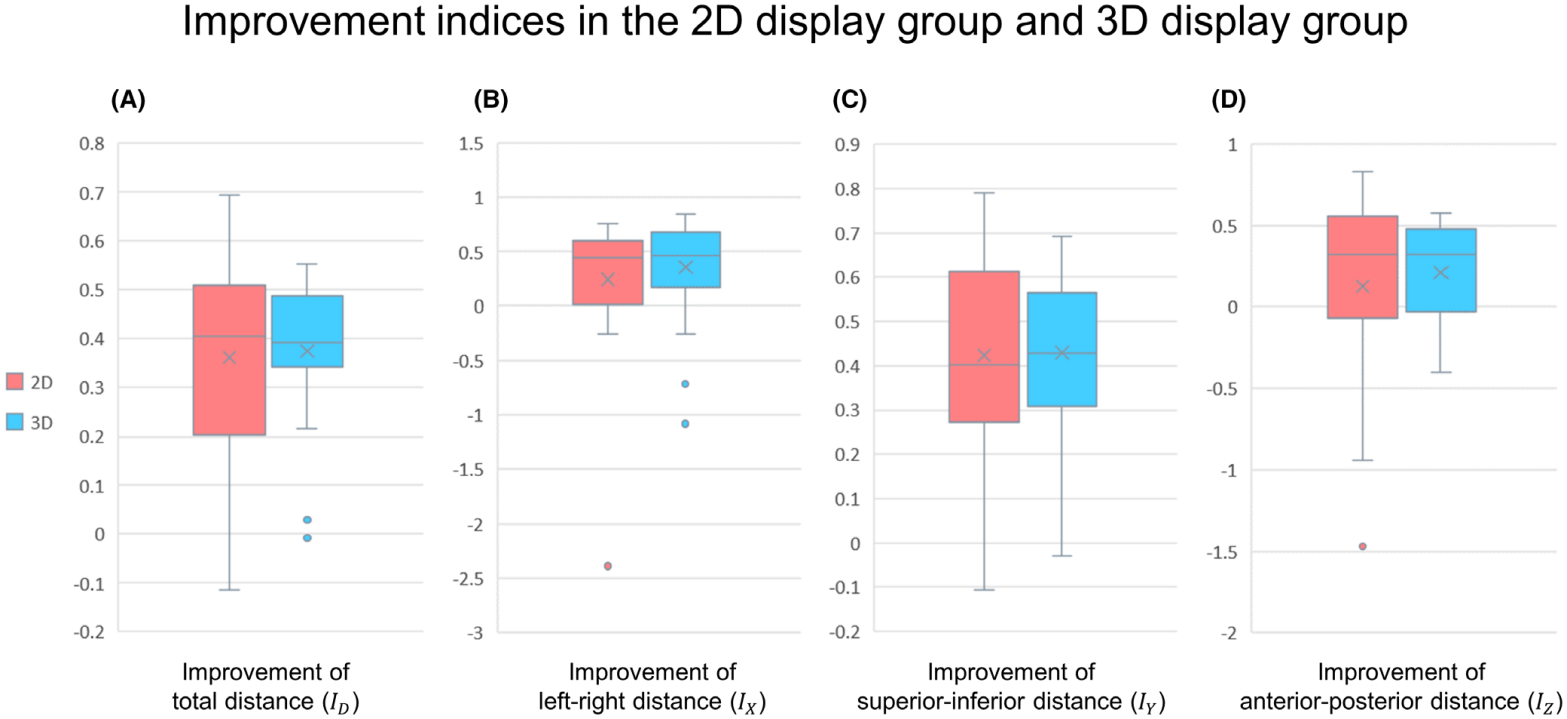
Comparative analysis of improvement indices of understanding of the spatial arrangement of anatomical structures between the 2D and 3D display groups. The boxplots present the statistical analysis of students' understanding of anatomical structures' the spatial arrangement before and after learning, with the mean values and standard deviations reported in the study. While both display methods showed improvements, the 3D display group exhibited less variability in the results, suggesting a more consistent understanding across individuals. This is supported by the smaller standard deviations for total distance, left–right, and anterior–posterior improvements, although no significant differences in mean values were observed. (A) Boxplot illustrating the improvement in total distance ($I_{D}$) in both the 2D (red) and 3D (blue) display groups, representing the change in participants' accuracy regarding the placement of anatomical structures in the 3D space from pre‐ to post‐learning evaluation. (B) Boxplot displaying the improvement in left–right distance ($I_{X}$) for both groups, indicating the reduced deviation of the placement of the anatomical structures along the *x*‐axis. (C) Boxplot demonstrating the improvement in the superior–inferior distance ($I_{Y}$) for both groups, reflecting the change in vertical positioning accuracy of the anatomical structures. (D) Boxplot depicting the improvement in anterior–posterior distance ($I_{Z}$) for both groups, signifying the participants' increased accuracy in positioning the anatomical structures along the depth axis.

Regarding the improvement index $I_{D}$, the mean was 0.37±0.21 and 0.39±0.14 for the 2D and 3D display groups, respectively, indicating no significant difference between the groups. However, the standard deviation was smaller in the 3D display group, indicating less variability (Figure 5A). A one‐sided *F*‐test, conducted to compare the variance, found that the variance in the 3D display group was significantly smaller (p=0.022) (Table 2). Similar to the findings for $I_{D}$, there was no difference in the means for $I_{X}$ and $I_{Z}$, but the standard deviation was significantly smaller in the 3D display group (Figure 5B,D; Table 2). For $I_{Y}$, both the mean and the standard deviation showed no difference between the groups (Figure 5C).

## DISCUSSION

This study developed 3D anatomy learning materials compatible with 3D spatial reality tabletop displays and used a 3D puzzle‐like method to directly assess and compare (vs. 2D displays) their effectiveness in enhancing understanding of the spatial arrangement of anatomical structures of students regarding the arrangement of anatomical structures. Although the use of 3D displays did not lead to a significant increase in average comprehension of these spatial arrangements compared with the use of 2D displays, it significantly reduced the variation in medical students' understanding of the spatial arrangement of anatomical structures. This suggests that 3D displays can provide a more universal learning experience across a wider range of students, compared to 2D displays. Furthermore, the 3D puzzle‐like evaluation method used in this study configures a novel approach for directly assessing participants' comprehension of anatomical structures' spatial arrangement, promising future applications in anatomy education and learning effectiveness evaluation.

In anatomy education, understanding the spatial arrangement of human body structures is crucial. Therefore, the anatomy knowledge that medical students are expected to acquire includes anatomical terminology, verbalized descriptions of structural features, and a direct understanding of the three‐dimensional arrangement of anatomical structures, which is an equally important component of anatomy knowledge. To deepen this understanding of the spatial arrangement of anatomical structures, 3D models have been increasingly adopted. Research on the effectiveness of 3D model‐based anatomical education can be broadly divided into two categories: (1) those that compare the use of 2D (e.g., images and texts in anatomy atlases) and 3D objects as learning materials,^15^, ^21^, ^23^, ^34^ and (2) those that compare the use of 2D and 3D displays for showing 3D object materials.^39^, ^45^ For the former type, even if the material is in 3D, it may still be presented on a conventional flat monitor or other 2D displays, potentially not fully leveraging the advantages and learning effectiveness of the 3D materials. The latter type involves methods using 3D display devices, such as stereoscopy,^34^, ^38^ augmented reality,^39^, ^45^, ^46^, ^47^ and VR,^33^, ^40^, ^48^, ^49^, ^50^ and compares learning outcomes related to using 3D displays with those related to 2D displays. Our study falls into the latter category, involving an RCT comparing the effectiveness of 3D educational materials presented in 2D versus 3D displays. However, it distinguishes itself from previous literature by the 3D display device used. Glasses‐free 3D displays, such as spatial reality tabletop 3D displays, enable stereoscopic vision without the need for special goggles, offering higher accessibility compared with 3D displays that require glasses. VR goggles can sometimes pose problems due to discomfort, pressure, or VR sickness.^45^, ^46^ In particular, mobile VR, which is relatively inexpensive, has been reported to cause significantly higher rates of side effects, such as blurred vision and loss of spatial orientation, compared to desktop VR.^47^ On the other hand, glasses‐free 3D displays avoid such issues, making them valuable devices that tackle the shortcomings of VR goggles. The fact that glasses‐free 3D displays are contactless and avoid learner isolation from the outside world also poses significant advantages for their use in medical education settings in which infection control and communication with others are emphasized.

While glasses‐free 3D technology offers significant advantages and high applicability, it has the limitation of not providing tactile input. This lack of tactile feedback is a limitation common to all digital learning materials, whether 2D or 3D. Traditional anatomy models remain attractive because they provide learners with tactile feedback. In recent years, advances in 3D printing technology have increased the flexibility of creating physical models, leading to their application in anatomy education. In an RCT comparing learning with 3D‐printed models and VR, using 3D‐printed models resulted in significantly higher test scores, suggesting that the absence of tactile feedback may limit the learning effectiveness of 3D visualization through VR.^48^ However, rather than choosing between these different learning methods or using them as alternatives, combining them may maximize their effectiveness. In fact, there have been attempts to develop integrated educational tools that combine 3D‐printed physical models with 3D digital materials to compensate for the lack of tactile feedback, a drawback of 3D digital learning materials.^49^, ^50^ Physical models that allow tactile interaction may be more advantageous for understanding the shape of individual structures. However, the strength of 3D digital visualization lies in its ability to float structures in space and reproduce their spatial relationships without the constraints of gravity, such as models falling over. Furthermore, the digital content allows for the quantitative assessment of learning effectiveness, such as through the puzzle‐like method introduced in this study, providing learners with objective feedback on their learning outcomes. The combination of glasses‐free 3D displays with 3D‐printed physical models could help mitigate the lack of tactile feedback while retaining the strengths of 3D digital visualization. This integrated approach holds potential for improving learning effectiveness and represents a promising avenue for future exploration.

As mentioned, the evaluation method used for assessing learning effectiveness in the current RCT is distinctive. Previous RCTs examining the efficacy of 3D educational materials and displays primarily utilized paper tests involving multiple‐choice and word‐answer questions.^15^, ^16^, ^23^, ^38^, ^39^, ^45^ These methods are relevant for various current educational practices as most universities evaluate student understanding of topics through such tests; concomitantly, they often rely on the memorization of anatomical terms and the verbalization of spatial relationships, thus not directly evaluating students' actual understanding of anatomical structures' spatial arrangement regarding the arrangement of organs and blood vessels. Bogomolova et al. pointed out that multiple‐choice questions can be answered correctly through guessing, even without an understanding of spatial arrangements, thus being potentially ineffective for evaluating the effectiveness of 3D learning materials presented in 3D displays.^45^ Specifically, the advantage of 3D learning materials presented in 3D displays lies in their ability to visualize 3D arrangements, including depth, to facilitate user understanding, which traditional assessment methods might not adequately reflect. Therefore, the inconsistency in the outcomes of the effectiveness of 3D learning materials in previous RCTs might partly be due to the limitations of these traditional assessment methods.^15^, ^16^, ^20^, ^21^, ^23^, ^33^, ^34^, ^38^, ^39^, ^45^

The 3D puzzle‐like method established and used in this study allows for a direct assessment of learners' comprehension of the spatial arrangement of anatomical structures and does not necessarily require the processes of memorizing anatomical terms or verbalizing spatial relationships. That is, instead of requiring medical students—who will become medical doctors performing physical examinations, ultrasound examinations, and surgeries—to choose, for example, the correct option from five options explaining anatomy, it requires the grasping of where each anatomical structure is located in the human body. This renders the 3D puzzle‐like method a groundbreaking approach for directly measuring the degree of anatomical understanding of students, a skill that is actually required of medical students and doctors in clinical settings. This study does not assess whether participants memorized anatomical structures' names or could describe their features in text, as is common in traditional paper tests. As mentioned, the spatial arrangement of anatomical structures is a critical component of anatomy knowledge. The learning tool used in this study was not designed to help memorize the names of structures but to acquire new anatomical knowledge about the three‐dimensional shapes of organs and their spatial arrangement. Accordingly, the evaluation methods were tailored to this focus. The challenge of relying on 2D paper tests for evaluation, even as educational methods are transitioning to 3D, has been noted in previous research. For instance, Bogomolova et al. developed and reported an evaluation method using AR applications to address this issue.^40^ Similarly, our 3D puzzle‐like method is expected to enhance the alignment between educational tools and evaluation methods. This innovative method could also be useful for anatomy learning improvement. Indeed, training with puzzle games has been reported to enhance spatial abilities.^40^ An educational session using 3D anatomical puzzles created with 3D printing demonstrated a statistically significant increase in learners' confidence in their anatomical knowledge. This suggests that the game‐like nature of puzzles can positively contribute to learning outcomes.^45^ These discussions imply that the 3D puzzle‐like method has the potential to be utilized in both the learning and assessment phases of medical and anatomical education.

In this RCT, while the average learning comprehension between the 3D and the 2D display groups did not differ significantly, the variability was significantly reduced in the 3D display group. To simplify the operation of the software for students, the learning phase allowed the 3D objects to be rotated only horizontally. Significant differences in variability between the two groups were observed in the left–right and anterior–posterior axes, which are visualized in 3D through horizontal rotation, but not in the superior–inferior axis. These results suggest that the reduced variability was influenced by the 3D display, potentially supporting a more consistent learning experience among students. The inference here is that a 3D display may help reduce differences in learning outcomes and create a more accessible learning environment for a wider range of students. Indeed, scholars have remarked that the educational effectiveness of anatomy education resources is not always uniform, and the necessity of considering individual learner characteristics when developing and introducing new learning materials should be acknowledged.^38^ Anatomical learning materials in 3D presented with 3D displays may be universally applicable and provide a more inclusive learning environment by securing more favorable learning outcomes irrespective of individual student characteristics.

This study was designed based on constructivism, dual coding theory, and cognitive load theory. Constructivism is a theory that posits that learners construct deep understanding by connecting new knowledge to their existing knowledge frameworks. This theory is particularly important in anatomy, where visual and spatial comprehension is essential. The 3D display used in this study provided a learning environment grounded in constructivist principles, enabling learners to explore and understand the spatial relationships between organs and structures at their own pace, facilitating the construction of knowledge. Dual coding theory suggests that learning effectiveness is enhanced by combining visual and verbal information. The learning materials on the 3D display integrated the three‐dimensional visual representation of anatomical structures with complementary verbal information (e.g., the names of structures presented as anatomical terms), effectively promoting the learners' information processing. Such multimodal integration has been shown to improve novice learners' interpretation of anatomical structures, particularly in cross‐sectional anatomy.^51^ Cognitive load theory aims to maximize learning efficiency by appropriately managing the load on the learner's working memory. By using the 3D display, the study addressed the challenges posed by flat images and texts, which can make it difficult to understand complex spatial structures. The visual supplementation provided by the 3D display reduced the cognitive burden on learners and created an environment that allowed them to focus on key information. This study applied these theoretical foundations, incorporating insights from existing research and educational theories, to evaluate the effectiveness of an anatomy learning tool that utilizes 3D displays.

## LIMITATIONS

This study has several limitations. First, the research is based on a population limited to medical students at Tokyo Medical and Dental University, which restricts the generalizability of the findings with regard to a broader population. Second, this study was limited to specific anatomical areas (i.e., abdominal organs and blood vessels); thus, the effectiveness of 3D learning materials for other anatomical regions must still be explored. Third, the study only evaluated post‐learning outcomes immediately after the learning process; that is to say, the long‐term impact on learning and its application in clinical practice is yet to be determined. Fourth, the study did not assess students' spatial abilities, precluding the examination of the relationship between spatial abilities and learning effectiveness. Previous studies have shown that spatial ability can influence anatomy learning performance and may interact with different instructional formats or visualization tools.^52^, ^53^, ^54^, ^55^, ^56^, ^57^, ^58^, ^59^, ^60^, ^61^ Future studies should consider evaluating learners' baseline spatial ability to better understand individual variability in response to 3D and 2D learning environments. Finally, this study used an RCT to assess the effectiveness of 3D learning materials but lacked qualitative data. Future research is invited to explore the broader applicability and long‐term effects of 3D learning materials in anatomical education, incorporating Likert‐type surveys, free‐text questionnaires, and focus group data to capture deeper perceptions.

## CONCLUSION

The 3D anatomical learning materials for abdominal organs and blood vessels developed in this study, compatible with glasses‐free 3D displays, are thought to offer significant advantages for medical education because of their accessibility and the benefit of non‐reliance on glasses/headsets. The 3D puzzle‐like method established to assess the learning effect of these 3D anatomical learning materials can facilitate the direct evaluation of the understanding of the spatial arrangement of anatomical structures in medical students. It can also aid in anatomy learning training. Using this method to assess the learning effectiveness of 3D learning materials presented through 3D displays revealed that 3D displays could reduce individual differences in learning effectiveness (vs. 2D displays). The results of this study suggest that using 3D displays can provide a more universally effective learning environment, enabling students with diverse characteristics to fully benefit from the learning effects of 3D educational materials. In contrast, using 2D displays may not achieve the same level of adaptability. Presenting 3D learning materials usng 3D displays is advantageous in supporting students with varying needs, providing a more inclusive and effective learning experience.

## AUTHOR CONTRIBUTIONS

**Satoru Muro:** Conceptualization; data curation; formal analysis; investigation; methodology; project administration; resources; software; supervision; validation; visualization; writing – original draft; writing – review and editing. **Keisuke Miyosawa:** Data curation; formal analysis; investigation; methodology; project administration; resources; software; validation; visualization; writing – original draft; writing – review and editing. **Kumiko Yamaguchi:** Conceptualization; data curation; formal analysis; investigation; methodology; project administration; resources; software; supervision; validation; visualization. **Kentaro Okamoto:** Formal analysis; investigation; methodology; project administration; resources; software; supervision; validation; visualization; writing – review and editing. **Shota Okamoto:** Formal analysis; investigation; methodology; project administration; resources; software; validation; visualization; writing – review and editing. **Tomoki Itamiya:** Data curation; formal analysis; investigation; methodology; project administration; resources; software; supervision; validation; visualization; writing – review and editing. **Keiichi Akita:** Conceptualization; formal analysis; methodology; resources; software; supervision; validation; visualization; writing – review and editing.

## FUNDING INFORMATION

No funding was received to carry out this study.

## CONFLICT OF INTEREST STATEMENT

The authors declare no conflict of interest.

## ETHICS APPROVAL STATEMENT

Study approval was obtained from the Board of Ethics at the Tokyo Medical and Dental University (approval number: C2023‐024). All participants provided informed consent prior to their inclusion in the study.

## Data Availability

The data supporting this study's findings are available from the corresponding author upon reasonable request. However, due to privacy and ethical restrictions, the data are not publicly available.
